# 1.8 V Aqueous Symmetric Carbon-Based Supercapacitors with Agarose-Bound Activated Carbons in an Acidic Electrolyte

**DOI:** 10.3390/nano11071731

**Published:** 2021-06-30

**Authors:** Chih-Chung Lai, Feng-Hao Hsu, Su-Yang Hsu, Ming-Jay Deng, Kueih-Tzu Lu, Jin-Ming Chen

**Affiliations:** 1 National Synchrotron Radiation Research Center, Hsinchu 30076, Taiwan; ed0410aevm@gmail.com (C.-C.L.); hsu.fh@nsrrc.org.tw (F.-H.H.); hsu.sy@nsrrc.org.tw (S.-Y.H.); 2 Department of Applied Chemistry, Providence University, Taichung 43301, Taiwan

**Keywords:** hydrogen-evolution reaction, supercapacitor, agarose-bound activated carbon

## Abstract

The specific energy of an aqueous carbon supercapacitor is generally small, resulting mainly from a narrow potential window of aqueous electrolytes. Here, we introduced agarose, an ecologically compatible polymer, as a novel binder to fabricate an activated carbon supercapacitor, enabling a wider potential window attributed to a high overpotential of the hydrogen-evolution reaction (HER) of agarose-bound activated carbons in sulfuric acid. Assembled symmetric aqueous cells can be galvanostatically cycled up to 1.8 V, attaining an enhanced energy density of 13.5 W h/kg (9.5 µW h/cm^2^) at 450 W/kg (315 µW/cm^2^). Furthermore, a great cycling behavior was obtained, with a 94.2% retention of capacitance after 10,000 cycles at 2 A/g. This work might guide the design of an alternative material for high-energy aqueous supercapacitors.

## 1. Introduction

A supercapacitor (SC) is an energy-storage system that has attracted great research interest because of its superior properties of ultra-high density of output power, excellent cycling stability, and safety consideration compared with batteries [1,2,3], but the small energy density of a SC creates difficulty for widespread applications. In general, the energy density of a SC is determined by its specific capacitance and the corresponding operating voltage. The strategies to obtain high-performance devices depend on the optimization of these two parameters. Instead of increasing the device capacitance, increasing the maximum operating voltage of a SC might become the most promising approach to improve the performance of SCs [4]. By definition, a capacitor stores electrical energy through two mechanisms [1,5]: (i) electrostatic attraction of charged ions in electrical double layers (EDL) formed at the interface between the electrode and electrolyte; (ii) a pseudo-faradaic charge-transfer reaction between the electrode and electrolyte. At present, a commercial SC, classified as an electrical double-layer capacitor (EDLC) using activated carbon electrodes in an organic electrolyte, is mostly available on the market. The features of large surface area, effective chemical stability, and excellent electrical conductivity make carbon materials significant as electrode materials in energy storage [6]. In contrast, the large voltage window of an organic electrolyte (e.g., 2.5–2.7 V for tetraethylammonium tetrafluoroborate in acetonitrile), achieving an attainable energy density, matches the demands for commercial use [7]. There remain, however, serious drawbacks to be remedied, such as a limited rate of charge and discharge resulting from a smaller electrolyte conductivity and the requirement of a moisture-free environment to build devices to ensure the stability of organic electrolytes [8,9]. In general, the operating voltage of an aqueous electrolyte is limited to 1.23 V [2,10], capped by the appearances of the hydrogen-evolution reaction (HER) and the oxygen-evolution reaction (OER), despite being typically more conductive than an organic electrolyte. As a result, the operating voltage of an aqueous SC is normally below 1.2 V, in contrast with the operating voltage of an organic SC up to 3.0 V or greater, resulting in a smaller energy density than for an organic SC even though the former has a greater capacitance than the latter while employing the same electrode material [9].

To exploit the advantages of an aqueous SC, its voltage window must be extended without the appearance of the HER and OER, which lead to decomposition of the device electrolyte. Researchers previously found that the surface functionality of a carbon electrode material influences the overpotential of the HER and OER. Khomenko et al. introduced the concept of an asymmetric capacitor, which can be reversibly charged to 1.6 V, based on only activated carbon with varied surface functionalities using H_2_SO_4_ (1 M) as an electrolyte [11]. Similarly, Bichat et al. reported that a seaweed-derived carbon with large oxygen content as an electrode had a stable potential window of 2.4 V in a Na_2_SO_4_ electrolyte (0.5 M, pH-neutral) examined with cyclic voltammetry [12], but it could only be galvanostatically charged to a maximum of 1.6 V. Fit et al. recently utilized Li_2_SO_4_ (1 M, neutral medium) as an electrolyte to make a carbon–carbon SC with a voltage window of 2.2 V that exhibits a stable capacitance retention cycled with 1 A/g [13]. In addition, Gao et al. found further evidence that its maximum operating voltage is also restricted by an irreversible electro-oxidation process at the positive electrode through a temperature-programmed desorption analysis of the electrodes after long-term cycling [14]; they let a SC cycle stably up to 1.9 V using a Li_2_SO_4_ electrolyte. Both studies indicated that neutral electrolytes provide wider potential windows for carbon-based symmetric SCs. Another strategy can utilize the phenomenon that the potentials of both OER and HER shift with pH according to the Pourbaix diagram of water and the Nernst equation, that is, assembling the SCs with the positive and negative electrodes operating in decoupled acidic and alkaline electrolytes, respectively, which increases the stable cell voltage to about 1.8 V. An ion-exchange membrane assembled within the cell is required, however, to maintain the pH of each electrolyte separately [15].

Researchers around the world have devoted much effort to develop an aqueous SC with a large energy density [16,17], but the study regarding a symmetric carbon–carbon SC with a large energy density using an acidic electrolyte is still a major challenge. In previous work, the potential windows of the same kind of SCs typically reached up to 1.0 V because of the unstable cycling behavior of a SC in an acidic electrolyte. In our work, we developed a simple route utilizing an ecologically compatible polymer, named agarose [18,19,20], as a novel binder (instead of polyvinylidene fluoride, PVDF, or polytetrafluoroethylene, PTFE) to fabricate an active carbon–carbon symmetric aqueous SC. A wider potential window with an operating voltage of 1.8 V, attributed to a large overpotential for the hydrogen-evolution reaction (HER), was achieved with a carbon-fiber cloth (CFC) packaging the SC and with sulfuric acid as the current collector and aqueous electrolyte, achieving a great cycling stability with a 94.2% capacitance retention after 10,000 cycles at 2 A/g.

## 2. Materials and Methods

### 2.1. Material Preparation

As-purchased carbon-fiber cloths (CFCs, CeTech Co. Ltd., Taichung, Taiwan) were sliced into sizes of 15 × 25 mm and washed with acetone, ethanol, and deionized (DI) water several times, followed by drying for 30 min at about 80 °C. Activated carbon powder (AC powder, NORIT SA 2, ACROS Organics, Ward Hill, MA, USA) was first stirred in H_2_SO_4_ solution (2 M) for 30 min at 60 °C to modify its surface functionality [11], followed by extensive rinsing with DI water until a pH near 7. Before use, the treated AC powders were dried in an oven overnight.

Dry-treated AC powder, agarose hydrogel, and carbon black (SuperP) of a particular mass ratio dispersed in DI water were prepared as a slurry named ACAGSpP. Agarose hydrogel was prepared on stirring agarose powder (UniRegion Bio-Tech, Hsinchu, Taiwan) in DI water (5–20 mass %) at 120 °C until a transparent gel solution was formed. Another slurry, named ACPVDFSpP, was prepared as a reference sample, which was basically composed of the same carbon powder, except that the binder and solvent were replaced with polyvinylidene fluoride (PVDF) and N-methyl-2-pyrrolidone (NMP), respectively. Both slurries were sonicated for at least 2 h before use. Briefly, the electrode was fabricated on pasting an active carbon slurry on a CFC, followed by drying in an oven at 85 °C overnight to remove residual solvent. In this study, we chose a mass loading of ~0.7 mg/cm^2^ and a thickness of the ACAGSpP electrode of ~100 µm to fabricate SCs for the following investigation.

### 2.2. Material Characterization

Morphological analysis of the as-prepared carbon electrodes was conducted with a field-emission scanning electron microscope (JEOL FE-SEM 7800F, Tokyo, Japan). The surface chemical species of the samples were analyzed with an X-ray photoelectron spectrometer (XPS, PHI Quantera SXM, Chanhassen, MN, USA). Raman spectra were recorded with a spectrometer (HORIBA, LabRAM, HR800, Kyoto, Japan) with a laser (514 nm) to evaluate the quality of the active carbon materials. The mass of a specimen was measured with an analytical balance (XSE105DU, Mettler Toledo, Greifensee, Switzerland, readability of 0.01 mg at maximum capacity of 41 g).

### 2.3. Electrochemical Measurements

All electrochemical tests were undertaken with a workstation (PGSTAT 128N, Metrohm Autolab, Utrecht, The Netherlands). Before examination of the performance of a SC, as-prepared pasted CFCs (exposed active area approximately 10 mm × 15 mm) were first soaked in H_2_SO_4_ aqueous electrolyte (1 M) for 24 h to ensure complete immersion of the electrolyte into an electrode. The potential window of a carbon electrode in an aqueous electrolyte was measured in a three-electrode cell: pasted CFCs as a working electrode and counter electrode; saturated calomel electrode (SCE) as a reference electrode. Symmetrical carbon–carbon SCs were assembled with two identical pasted CFCs as electrodes and H_2_SO_4_ (1 M) as an electrolyte. The specific capacitances (*C*_sp_, F/g) of the symmetric SCs derived from galvanostatic discharge curves were calculated based on the following equation [21],

(1) $$C_{sp}=\frac{I\times ∆t}{m\times \Delta V}$$

where *I*, ∆*t*, and ∆*V* represent the discharge current, interval for full discharge, and operating potential difference of a SC, respectively. *m* denotes the total net mass of active material contained in two CFCs. The corresponding energy density (*E*, W h/kg) and power density (*P*, W/kg) were calculated as follows [21]:

*E* = (½) *C*_sp_ Δ*V^2^* (2)

(3) $$P=\frac{E}{\Delta t}$$

where ∆*V* is the SC voltage and ∆*t* is the corresponding discharge period. It has been discussed that the areal capacitance (F/cm^2^), areal energy density (W h/cm^2^), and areal power density (W/cm^2^) are more reliable metrics of the performance for supercapacitances [22]. In this report, we use both units (such as F/g and F/cm^2^) for clarity. Electrochemical impedance spectra (EIS) were measured with a sinusoidal signal of 10 mV over a frequency range from 100 kHz to 10 mHz.

## 3. Results and Discussion

**Figure 1:**
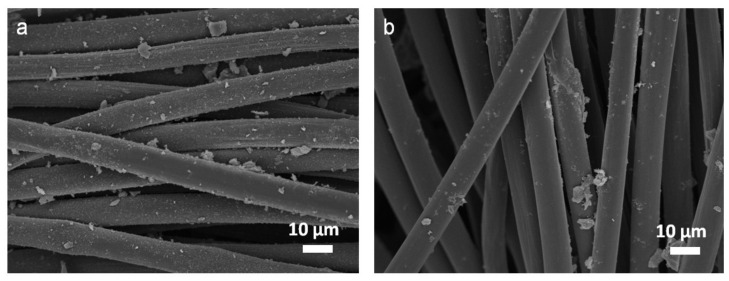
SEM images showing the surface morphologies of (**a**) ACAGSpP- and (**b**) ACPVDFSpP-decorated CFCs.

**Figure 2:**
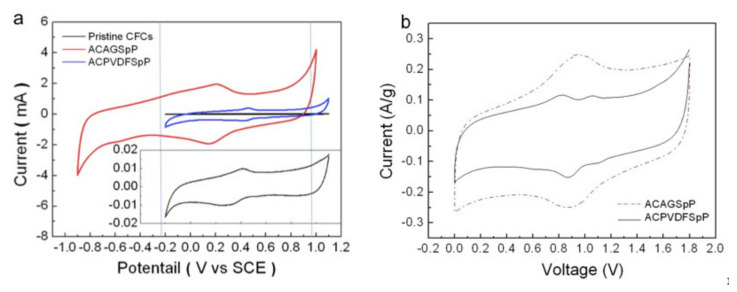
(color online) (**a**) CV curves show potential windows of ACAGSpP electrodes (red), ACPVDFSpP electrodes (blue), and pristine CFCs (black) in H_2_SO_4_ (1 M) at a scan rate of 10 mV/s; inset displays an enlarged plot of pristine CFCs; (**b**) CV curves obtained for symmetric aqueous SCs employing ACAGSpP electrodes or ACPVDFSpP electrodes at 10 mV/s with maximum voltage of 1.8 V.

The surface morphologies of AC-decorated as-prepared CFCs with two slurries are shown in Figure 1. Carbon fibers were cross-linked with each other as cloths, for which the AC powders were bound through two binders. AC powders were obviously deposited more homogeneously on CFCs bound with agarose (Figure 1a). Conversely, it seems that AC powders were somehow bound in the form of aggregated clusters onto CFCs with the use of PVDF as a binder (Figure 1b). The Raman spectra of these CFCs with or without pasting slurries all revealed common features of graphite with D-bands (~1350 cm^−1^) and G-bands (~1580 cm^−1^), as plotted in Figure S1. In general, the D-band and G-band can be attributed to the disorder (sp^3^) and graphitic (sp^2^) structure of carbon materials, respectively. The ratios of intensities of D-bands to G-bands were calculated to be about 1.01–1.05 for all CFCs, revealing the existence of disordered carbon (e.g. oxygen functional group) in the carbon materials. Figure 2a shows cyclic voltammograms (CV) of a three-electrode cell with CFCs loaded with ACAGSpP and ACPVDFSpP, named the ACAGSpP electrode and ACPVDFSpP electrode, respectively, in acidic aqueous medium (1M H_2_SO_4_) with a scan rate of 10 mV/s. The capacitive behaviors, including the EDL and the pseudo-faradaic contribution through their surface oxygenated functionalities, were observed for carbon electrodes pasted with these two slurries. These redox signals were observed from about −0.2 to 0.4 V vs. SCE for ACAGSpP and 0.3 to 0.6 V vs. SCE for ACPVDFSpP. It is traditional to assess redox reactions of oxygenated functionalities (e.g., quinone-hydroquinone pairs) at the surface of activated carbons, generated by partial oxidation using a hot acid treatment [23,24]; the reactions are shown in Equations (4) and (5).

C–OH − H^+^ − e^−^ ↔ C = O (4)

COOH − H^+^ − e^−^ ↔ COO (5)

To investigate further the content of the surface functional groups on these CFCs, we applied XPS to characterize their surface chemical compositions. The C 1s spectra of CFCs of three kinds were fitted with signals at 284.4, 285.1, 286.1, 287.4, 289, 290.3, and 291 eV, which were attributed to carbon in C–C (sp^2^), C–C (sp^3^), C–O, C=O, O–C=O, and C–F groups, and π–π in aromatic rings, respectively. On normalizing the peak intensity of C–C (Figure S2 and Table 1), we clearly observed a greater composition of oxygenated functionalities for ACAGSpP, which is consistent with the fact that the pseudo-faradaic redox reaction contributes to an important extent to its overall capacitance (red curve in Figure 2a). The potential window of a cell employing ACPVDFSpP as an active material was examined to be about 1.0 V, from 0 to 1.0 V vs. SCE, which is smaller than the thermodynamic stability window of water (1.23 V). The standard potentials at which the HER and OER occur are marked with two vertical dashed lines according to the previous literature [12]. What is especially noteworthy is that an overpotential of 0.3 V for the HER was found for a SC containing ACAGSpP-decorated CFCs, of which the potential window was extended toward the cathodic direction, indicating a potential window of range from −0.8 to 0.9 V vs. SCE, 1.7 V in total. In addition, the hydrophobic nature of the PVDF binder somehow restrained the aqueous electrolyte from diffusing into pores inside the electrodes, leading to a smaller current output for ACPVDFSpP-loaded electrodes, recorded as a blue curve in Figure 2a. For comparison, the CV of aqueous cells with blank CFCs at a scan rate of 10 mV/s was also analyzed (black curve), and it is enlarged in the inset of Figure 2a, which indicates a stable potential window of about 1.1 V, ranging from −0.1 to 1.0 V vs. SCE. Its capacitance, calculated to be approximately 1/100 those for ACAGSpP-decorated CFCs, shows its excellent characteristic as a current collector. Due to the high resistance and easy cracking, the electrochemical behavior of the pure agarose-electrode could not be obtained in sulfuric acid electrolytes. Figure 2b shows CV curves of two electrode systems cycled to 1.8 V utilizing ACAGSpP-decorated CFCs and ACPVDFSpP-decorated CFCs at scan rate of 10 mV/s. We observe in Figure 2b that no apparent anodic or cathodic tail appeared at the edges of the CV curves resulting from the decomposition of the aqueous electrolyte for the ACAGSpP electrodes, but tails, especially that appearing at 1.45–1.8 V for the ACPVDFSpP electrodes, were consistent with our investigation with the three-electrode measurement (Figure 2a).

**Figure 3:**
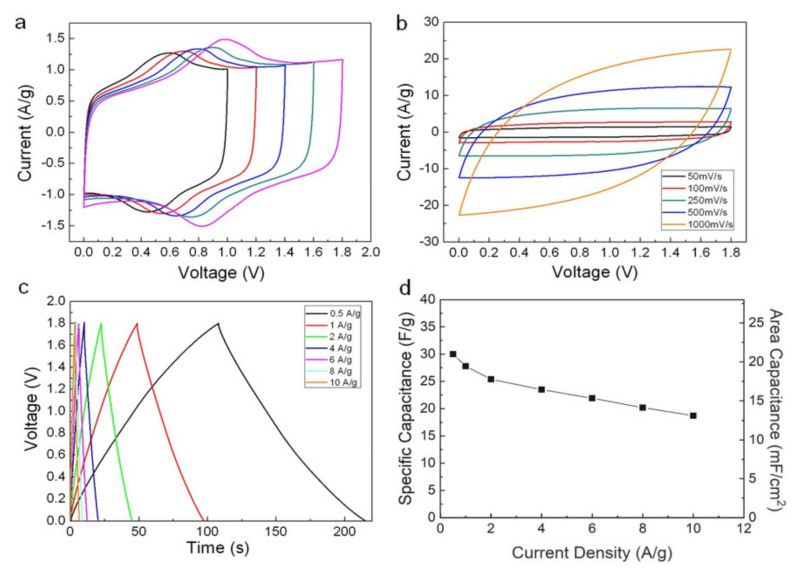
(color online) CV curves obtained for symmetric aqueous SCs employing ACAGSpP electrodes (**a**) with sweep rates of 50 mV/s at maximum voltages of 1, 1.2, 1.4, 1.6, and 1.8 V, respectively, and those (**b**) with sweep rates of 50, 100, 250, 500, and 1000 mV/s at voltages up to 1.8 V; (**c**) Galvanostatic charge and discharge curves, and (**d**) specific capacitances at various current densities.

The capacitance of the ACAGSpP electrodes was quantitatively larger than that of the ACPVDFSpP electrodes at a similar mass loading of the active materials. Accordingly, a less exposed active surface area is one reason for a poor electrochemical performance of ACPVDFSpP-decorated CFCs. About 80% of electrostatic charge storage was obtained at the electrode–electrolyte interface (EDLCs). Besides, a pseudo-faradic capacitive contribution of ~20% owing to their surface oxygenated functionalities was observed for carbon electrodes pasted with these two slurries. The ~80% electrostatic charge storage was calculated by the CV curves of ACAGSpP-loaded SCs with different scan rates in Figure 3b. The rectangularly shaped CV for EDLCs stems from the basic nature of the double-layer charge storage mechanism. In EDLCs, adsorption/desorption of ions at the electrode–electrolyte interface, in the absence of diffusion limitations, was almost instantaneous, so *dV/dt* was a constant. Therefore, EDLCs for practical applications require a power delivery over only a few seconds at a time. However, for pseudocapacitors, the charge is stored through Faradaic charge-transfer reactions when ions are electrochemically adsorbed onto the surface or near the surface of a material. At sweep rates over 500 mV/s, the b-value decreases to ~0.8 for the currents, indicating considerable battery-like behavior (pseudo-faradic capacitive). The relation between the current (*i*) and potential scan rate (*v*) can be generally expressed as Equation (6) [25]:

*i = av*^*b*^ (6)

with the value of *b* providing insight into the mechanism of charge storage. Both cells were successfully cycled at a voltage of 1.8 V, but there was a distinct capacitance decline of about 5% on the latter after being cycled to 1.8 V within just a few cycles, as plotted in Figure S3. It is especially noteworthy that there might be an irreversible reaction occurring on the ACPVDFSpP-loaded carbon electrode, accordingly damaging the surface of active sites for ions charging or discharging. This result indicates a less stable carbon material of ACPVDFSpP operating at a voltage 1.8 V. It should be pointed out that the electrochemical characterization of a single electrode was more important in “asymmetric supercapacitors (ASCs)”. A feasible approach to achieve a higher working voltage for aqueous electrolytes is to use two different electrode materials for the anode and the cathode. In this study, the symmetric supercapacitor was assembled by two identical ACAGSpP electrodes and sulfuric acid electrolytes. In the ACAGSpP electrode, most of the *C_sp_* was obtained from AC powder and carbon black (SuperP). The agarose was an electrode binder (or surfactant) in the ACAGSpP electrodes. Therefore, the electrochemical performance of one ACAGSpP electrode was replaced by the SCs with two ACAGSpP electrodes in this study.

In contrast, the ACAGSpP-loaded SCs showed steady CV curves with varied voltage windows ranging from 1 to 1.8 V at a scan rate 50 mV/s, as shown in Figure 3a (note that the data at each voltage were collected after measuring five cycles). Their CV curves exhibited a rectangular shape characteristic of additional cathodic or anodic signals located at nearly the center of each voltage scan, thus belonging to a pseudo-faradaic capacitive contribution. An EDL capacitive behavior was still retained, even cycled at a scan rate of 1 V/s (Figure 3b). Moreover, a varied mass loading influenced the capacitance of ACAGSpP electrodes: a greater mass loading typically results in a close-packed structure; thus, only a limited exposed surface area remains electrochemically active, leading to a decreased capacitance [26]. In our work, an identical trend was found for ACAGSpP-decorated CFCs, as plotted in Figure S4, for which the corresponding specific capacitances, calculated based on their CV with an operating voltage of 1.8 V, were 25.9, 25.4, 24.8 and 21.4 F/g (12.2, 17.8, 20.1, and 24.2 mF/cm^2^, respectively) at 50 mV/s for mass loadings of 0.47, 0.71, 0.81 and 1.13 mg/cm^2^, respectively. We chose a mass loading of about 0.7 mg/cm^2^ to fabricate SCs for the following investigation. The electrochemical performance of ACAGSpP-loaded SCs was further confirmed with galvanostatic charge and discharge (GCD) measurements, as plotted in Figure 3c, which shows symmetric GCD curves at various current densities, proving that the ACAGSpP displays a stable capacitive behavior and rapid charge and discharge capability with a voltage of 1.8 V. The quantities of specific capacitance derived from GCD curves at varied current densities are plotted in Figure 3d. The gravimetric capacitances were calculated to be 30.0, 27.8, 25.4, 23.5, 21.9, 20.2, and 18.7 F/g (21.0, 19.5, 17.8, 16.5, 15.3, 14.1, and 13.1 mF/cm^2^, respectively) at 0.5, 1, 2, 4, 6, 8, and 10 A/g, respectively.

**Figure 4:**
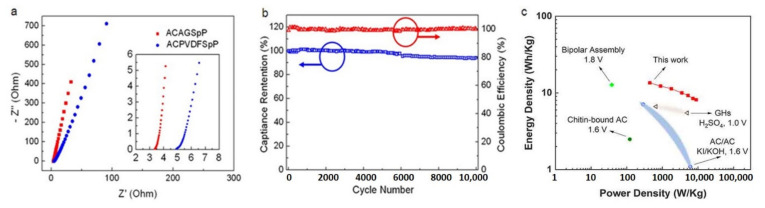
(color online) (**a**) Nyquist plot of symmetric aqueous SCs employing ACAGSpP or ACPVDFSpP electrodes; (**b**) cycling performance and coulombic efficiency of symmetric ACAGSpP SCs with operating voltage of 1.8 V at 2 A/g for 10,000 cycles, and (**c**) Ragone plot of gravimetric energy density versus gravimetric power density for ACAGSpP electrodes (this work) in comparison with other reported high-voltage symmetric aqueous carbon–carbon SCs.

To evaluate the ion diffusion of carbon composite electrodes in the aqueous electrolyte, we recorded electrochemical impedance spectra (EIS) with a frequency range from 0.01 Hz to 100 kHz. The Nyquist plots for ACAGSpP electrodes and ACPVDFSpP electrodes are displayed in Figure 4a. The Nyquist plot can be divided into the high- and low-frequency regions. In the high-frequency region, the semicircle corresponds to the charge-transfer resistance, whereas the slope of the straight line in the low-frequency region is related to the diffusion resistance in the electrode materials. Notably, these Nyquist curves show no obvious semicircles in the high-frequency region, indicating less charge-transfer resistance for both cells [27]. In the low-frequency region, the straight-line part for the ACAGSpP electrodes leans more toward the imaginary axis, indicating its superior capacitive behavior. The oblique line for the ACAGSpP electrodes is shorter than that for the ACPVDFSpP electrodes, implying that EDL formation was inhibited inside the micropores of the latter [28]. On extrapolating the vertical portion of the plots to the real axis, the equivalent series resistance (ESR) of ACAGSpP electrodes was calculated to be 3.33 Ω, which was slightly smaller than that of ACPVDFSpP electrodes (4.88 Ω). In a practical application, the long-term cycling performance of a SC is a critical parameter that must be examined. Previously, operation of a carbon-based aqueous SC in an acidic electrolyte was generally unstable, especially when cycled at a voltage exceeding 1.6 V. The irreversible reaction occurring at an activated loaded positive electrode was the main reason leading to a capacitance decline during cycling at a high voltage [14,29,30]. Nevertheless, in this work, the remarkable chemical stability of the ACAGSpP carbon composite in an acidic electrolyte was examined at a cycling voltage of 1.8 V. Figure 4b displays a long-term cycling test for symmetric ACAGSpP SCs at 2 A/g for 10,000 cycles. An excellent cycling stability was obtained, revealing a capacitance retention of 94.2% and a coulombic efficiency of nearly 100% after 10,000 cycles. On putting the first five and last five cycles of a long-term cycling test into the same plot, in Figure S5, the GCD curves showed both symmetric and reversible curves before and after the test, with the average specific capacitance for each five cycles calculated to be 25.4 F/g (17.8 mF/cm^2^) and 23.9 F/g (16.7 mF/cm^2^). To estimate further the potential of aqueous symmetric ACAGSpP SCs, we plotted Ragone plots of energy density versus power density of our ACAGSpP carbon electrode in Figure 4c. On considering the entire mass of active materials on two electrodes, a maximum energy density of 13.5 W h/kg (9.5 µW h/cm^2^) was delivered at a power density of 450 W/kg (315 µW/cm^2^), and 8.4 W h/kg (5.9 µW h/cm^2^) at a power density of 9.1 kW/kg (6398 µW/cm^2^). With the advantage of a wide voltage window of ACAGSpP electrodes in an acidic aqueous electrolyte, the obtained energy density was enhanced to 13.5 W h/kg (9.5 µW h/cm^2^), which is twice that of the commercial activated carbon SCs (5–7 W h/kg), even storing four times that of alkaline aqueous AC/AC SCs (3–4 W h/kg) [9]. The performance of the ACAGSpP-decorated SCs, together with previous reports for comparison, is listed in Table 2 [31,32,33,34]. From this table, in this work, the potential window using agarose as a binder is larger than those of the previous studies [30,31,32,33]. In comparison with novel carbons such as graphene-based materials, the energy density of our ACAGSpP-loaded SCs is significantly larger those of symmetric aqueous SCs using self-assembled graphene hydrogels (SGH) (e.g., 5.1 W h/kg at 700 W/kg) [31] and graphene hydrogels (HG) [32] (see Table 2). According to our results, the performance of the ACAGSpP-decorated SCs is better than that of the PVDF-binder SC (more than 30% increased, i.e., 30.0 F/g in this work and 20.2 F/g for the latter). Besides, according to the results from Béguin’s group, the performance of activated carbon symmetric capacitors based on two commonly used PVDF and PTFE as binders in an aqueous electrolyte only reached up to 1.6 V [35]. The PTFE-based and PVDF-based carbon electrodes exhibited capacitance values with only a 10% difference [35]. It also shows that agarose could serve as a novel binder fabricating activated carbon supercapacitors. This clearly indicates that the larger potential window using agarose as a binder is significantly improved over the corresponding value of energy density of a ACAGSpP-loaded SC. Accordingly, in this study, we focused on the improvement of the potential window and the maintenance of its capacitance by using agarose as a binder, thus further increasing the energy density.

## 4. Conclusions

We developed a simple approach utilizing agarose, an ecologically compatible polymer, as a novel binder (instead of PVDF or PTFE) to fabricate an activated carbon supercapacitor, enabling a wider potential window attributed to a greater HER overpotential of agarose-bound activated carbon in sulfuric acid. We fabricated 1.8 V symmetric aqueous SCs featuring agarose-bound activated carbon onto CFCs in H_2_SO_4_ electrolyte (1 M). A potential window of about 1.7 V (−0.8 to 0.9 V vs. SCE) was achieved at a scan rate of 10 mV/s investigated with three-electrode cyclic voltammetry, which resulted from a large HER overpotential of an ACAGSpP electrode. In contrast, a cell with the same active material but utilizing PVDF as a binder revealed a narrow potential window, approximately 1.0 V (0 to 1.0 V vs. SCE) at 10 mV/s. Additionally, extra cathodic-anodic signals were observed for ACAGSpP electrodes in H_2_SO_4_ (1 M), thus enhancing its capacitance through storing electrical energy by pseudo-capacitive reactions. The SC employing two identical ACAGSpP electrodes in a symmetric set was cycled up to 1.8 V galvanostatically, achieving a specific capacitance of 30.0 F/g (21.0 mF/cm^2^) at 0.5 A/g. EIS measurements revealed a compatible ESR for ACAGSpP electrodes in H_2_SO_4_ (1 M). Furthermore, ACAGSpP electrodes showed a great long-term stability (94.2% of capacitance retention after 10,000 cycles) cycled to 1.8 V at 2 A/g. The cell with ACAGSpP electrodes (13.5 W h/kg or 9.5 µW h/cm^2^) had twice the energy density of traditional commercial EDLC (5–7 W h/kg). Furthermore, this designed technique might be universal for the assembly of various porous materials as electrodes for SCs. For example, an enhanced capacitance or energy density might be obtained on using other carbon materials (e.g., graphene, carbon nanotube, and carbide-derived carbon) or those with optimized surface areas and distributions of pore sizes. This work provides an alternative material design featuring the advantages of low cost and simple manufacture for aqueous symmetric carbon SCs with a large operating voltage.

## Figures and Tables

**Table 1:** XPS curve-fitted results of C 1s spectra.

Sample	C–C (sp^2^)	C–C (sp^3^)	C–O	C=O	COOH	C–F	π-π*
Pristine CFCs	61.64	13.82	10.73	4.46	3.82	x	5.53
ACAGSpP	57.32	11.13	15.99	6.85	4.43	x	4.28
ACPVDFSpP	62.71	13.66	10.19	4.03	2.46	3.59	3.36

**Table 2:** Comparison of carbon-based symmetric supercapacitors.

Electrode Material	Electrolyte	Potential Windows (V)	Capacitances (F/g)	Energy Density (Wh/Kg)	Power Density (W/Kg)	Cycle Stability	Ref.
Self-assembled Graphene Hydrogels	1 M H_2_SO_4_	1.4	87.6 (at 1 A/g)	5.1	700	N.A.	30
Graphene Hydrogels	1 M H_2_SO_4_/PVA	1	186 (at 1A/g)	0.61	670	91.6% after 10k cycles	31
Graphene Foam/PVA	1 M Na_2_SO_4_	1.6	65 (at 0.2 A/g)	12	400	100% after 3000 cycles	32
HPCRs *	6 M KOH/PVA	1	48.7 (at 0.2A/g)	6.77	100	81% after 10k cycles	33
ACAGSpP	1 M H_2_SO_4_	1.8	30.0 (at 0.5A/g)	13.5	450	94.2% after 10k cycles	This work

* hierarchical porous carbon nanorods.

## Data Availability

Data are contained within the article.

## Supplementary Materials

The following are available online at https://www.mdpi.com/article/10.3390/nano11071731/s1, Figure S1. Raman spectra of as prepared electrodes of CFP only (black), ACAGSpP (red), and ACPVDFSpP (blue) (Probed by 532 nm laser), Figure S2. Fitted XPS spectra in C1s of (**a**) CFP-only, (**b**) ACAGSpP and (**c**) ACPVDFSpP electrodes. The peaks’ intensities are normalized according to that of C–C sp^2^ (284.4 eV) in each sample. Figure S3. CV curves of ACPVDFSpP-decorated CFCs at initial (brown line) and after operation for several cycles (black dashed line). [Scan rate is 50 mV/sec], Figure S4. Specific capacitance of the ACAGSpP electrodes at various mass loading on CFCs. [Note] The active area is fixed to be 1 x 1.5 cm^2^ for all the cases, Figure S5. The first five (blue solid line) and last five (black dashed line) galvanostatic charge/discharge curves of symmetric aqueous SCs employing ACAGSpP electrodes at 2 A/g for 10,000 cycles.
