# Supplementary material for: 1.8 V Aqueous Symmetric Carbon-Based Supercapacitors with Agarose-Bound Activated Carbons in an Acidic Electrolyte
Source: Nanomaterials (Basel). 2021 Jun 30;11(7):1731. doi: 10.3390/nano11071731 (PMC8308127; doi:10.3390/nano11071731)
Supplement: Supplementary file 1 [file nanomaterials-11-01731-s001.zip › nanomaterials-1265309-supplementary.pdf]

nanomaterials

Supplementary Materials: 1.8 V Aqueous Symmetric Carbon-Based Supercapacitors with Agarose-Bound Activated Carbons in an Acidic Electrolyte

Chih-Chung Lai [1,†], Feng-Hao Hsu [1,†], Su-Yang Hsu [1,†], Ming-Jay Deng [2,*], Kueih-Tzu Lu [1,*] and Jin-Ming Chen [1,*]

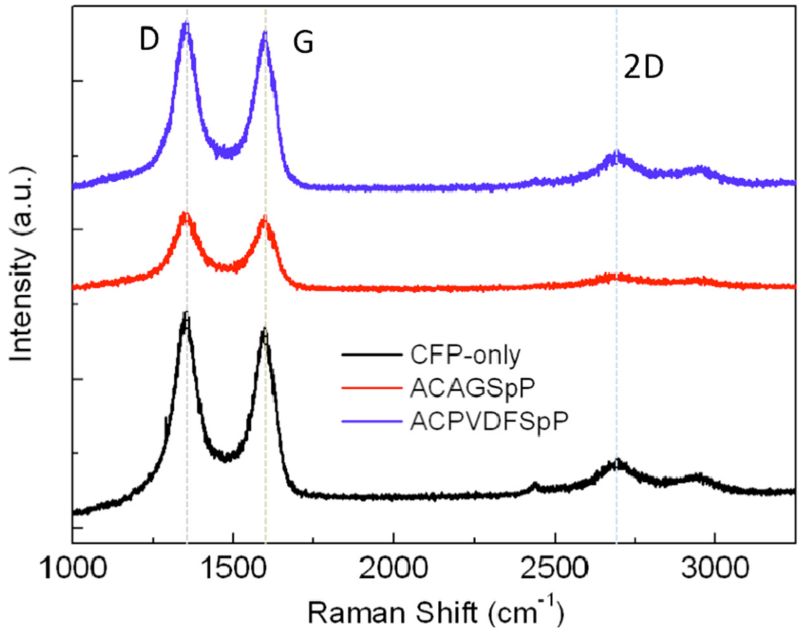

Figure S1. Raman spectra of as-prepared electrodes of CFP-only (black), ACAGSpP (red), and ACPVDFSpP (blue) (Probed by 532 nm laser).

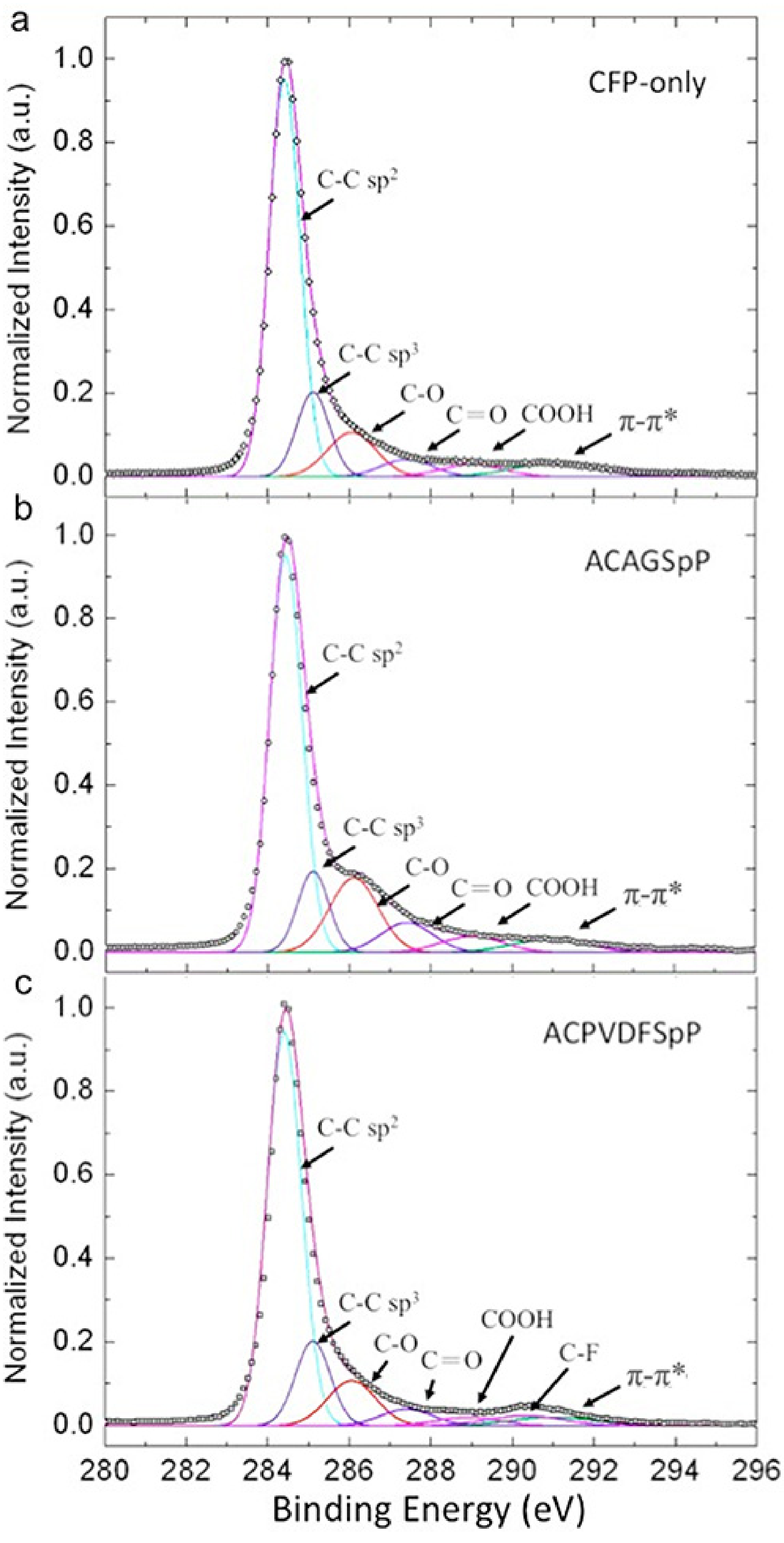

Figure S2. Fitted XPS spectra in C1s of (**a**) CFP-only, (**b**) ACAGSpP and (**c**) ACPVDFSpP electrodes. The peaks' intensities are normalized according to that of C–C sp^2 (284.4 eV) in each sample.

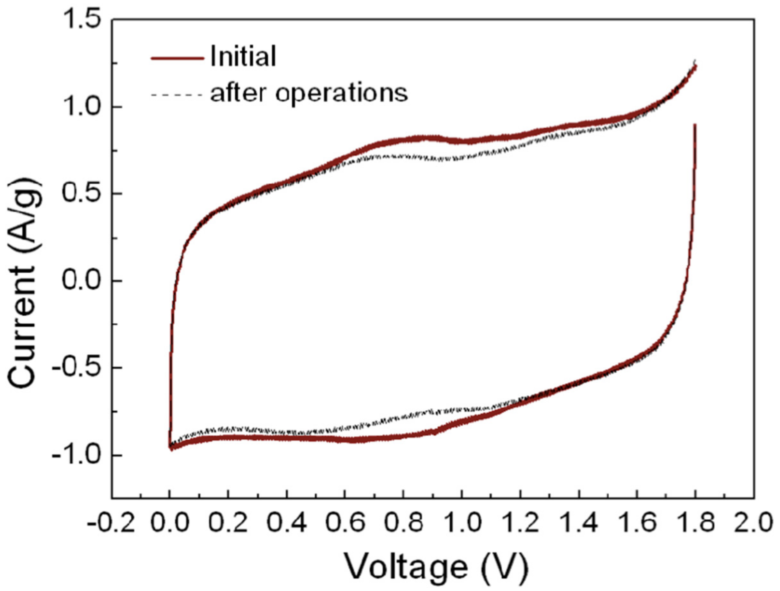

Figure S3. CV curves of ACPVDFSpP-decorated CFCs at initial (brown line) and after operation for several cycles (black dashed line). [Scan rate is 50 mV/sec].

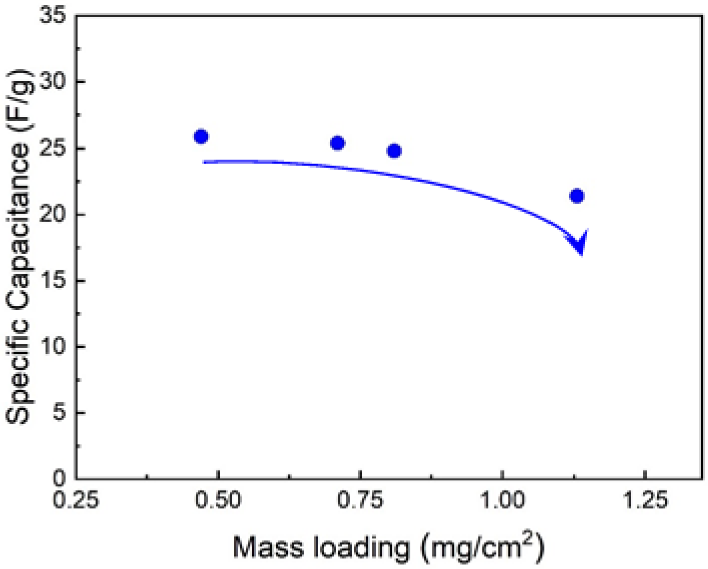

Figure S4. Specific capacitance of the ACAGSpP electrodes at various mass loading on CFCs. The active area is fixed to be 1 x 1.5 cm^2 for all the cases.

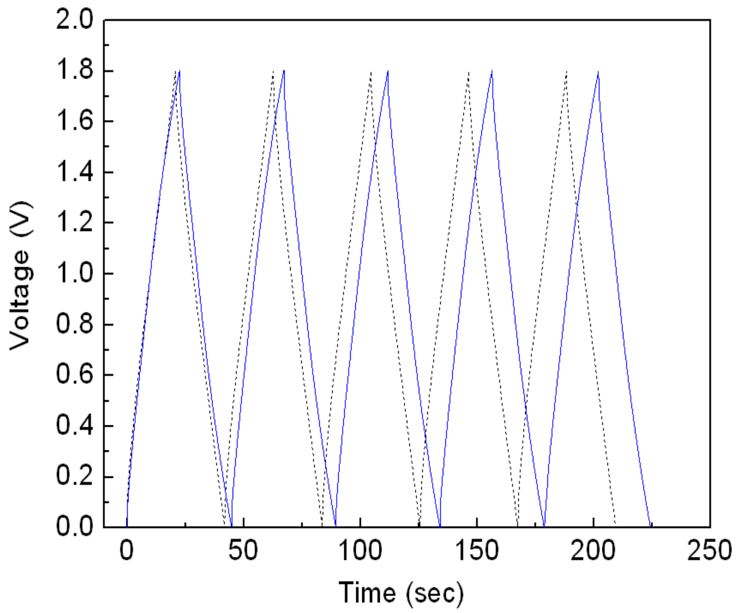

Figure S5. The first five (blue solid line) and last five (black dashed line) galvanostatic charge/discharge curves of symmetric aqueous SCs employing ACAGSpP electrodes at 2 A/g for 10,000 cycles.
